# Devoted or negotiated routes of adherence: Narratives of patients with obstructive sleep apnoea using a continuous positive airway pressure device

**DOI:** 10.1002/nop2.325

**Published:** 2019-07-01

**Authors:** Margareta Møkleby, Anne Marit Mengshoel

**Affiliations:** ^1^ Department of Interdisciplinary Health Sciences, Faculty of Medicine, Institute of Health and Society University of Oslo Oslo Norway; ^2^ Pediatric and Adult Sleep Disorder Clinic, ENT Department Lovisenberg Diaconal Hospital Oslo Norway

**Keywords:** adherence, adjustment, continuous positive airway pressure, interview, long‐term illness, narratives, nursing, obstructive sleep apnoea, patient experiences, qualitative

## Abstract

**Aims:**

To explore the narratives of patients about receiving a diagnosis of obstructive sleep apnoea and using a continuous positive airway pressure device.

**Design:**

A qualitative design with a narrative approach.

**Methods:**

Participants with personal experience of using a continuous positive airway pressure device were recruited through purposive sampling. Two women and five men were interviewed in 2017. The data material was subjected to a narrative analysis.

**Results:**

Two storylines portraying two different trajectories of continuous positive airway pressure use were identified: “A route of devotion” reflects patients experiencing immediate health benefits, which lead to committed use. “A route of negotiations” is characterized by more irregular use, and the perceived benefits were less evident than for those displaying a devoted route. Individuals adjust to a continuous positive airway pressure device differently based on their prior and present life situation and whether use of the device is experienced as an opportunity to regain ordinary life or as an obstacle to maintaining ordinary life.

## 1 INTRODUCTION

Obstructive sleep apnoea (OSA) is one of the most common sleep disorders. The prevalence in the adult population worldwide is 3%–7% and is more prevalent among men than women (Punjabi, 2008). In OSA, a collapse of the upper airways occurs during sleep, which often leads to poor sleep and excessive daytime sleepiness (Weaver & Sawyer, 2010). Not all patients experience such symptoms (Campos‐Rodriguez, Martinez‐Alonso, Sanchez‐de‐la‐Torre, & Barbe, 2016), but untreated OSA can nevertheless have an impact on one's general health and increase the risk of future abnormal glucose metabolism and cardiovascular diseases (Punjabi, 2008). As snoring is common with OSA, sleeping partners also can suffer from poor sleep. Additionally, untreated OSA heightens the risk of poorer waking function and is associated with a higher risk of road traffic accidents (Iversen, Broström, & Ulander, 2018).

The gold standard for treatment of OSA is continuous positive airway pressure (CPAP). The CPAP device, which is about the size of a small radio, is placed on the nightstand and attached to the patient with a tube and a facial mask (Shapiro & Shapiro, 2010). Its effectiveness in preventing apnoeas is well established, but as CPAP use tends to be low, its total effectiveness is limited (Weaver & Sawyer, 2010).

## 2 BACKGROUND

Suffering from a long‐term condition often includes treatment with either medication, medical home equipment or lifestyle changes. In general, the World Health Organization (WHO) (2003) points out that poor adherence is a significant challenge in lifelong treatments, as about 50% of people with long‐term conditions do not adhere to prescribed therapies. Among CPAP users, Weaver and Sawyer (2010) point to how estimates of adherence range from 30%–60% and are often based on measurements downloaded from the device indicating the number of hours per night the machine was used. Weaver and Sawyer (2010) further underline that the cut‐off for defining “how much CPAP use equates to adherence” (p. 247) is hard to determine. Studies have applied various cut‐offs, both when reporting on patients’ adherence and when evaluating interventions implemented to promote CPAP adherence, but no universal standard exists.

Continuous positive airway pressure treatment can prevent apnoeas, but does not cure OSA, and for some, the treatment might be lifelong. In clinical as well as research settings, poor CPAP adherence has been of significant concern and studies have been conducted to determine how best to predict and promote CPAP use. No single factor, such as a patient characteristic (e.g. age, sex, marital status), disease characteristic (e.g. disease severity) or technological factor (e.g. choice of mask), has been identified as a significant predictor of CPAP adherence (Weaver & Sawyer, 2010). The conclusion of many studies has rather highlighted how CPAP adherence is the result of several intervening elements, such as those above mentioned, in addition to experiencing social support and strong collaboration with healthcare personnel (Shapiro & Shapiro, 2010; Weaver & Sawyer, 2010). Educational, supportive and behavioural interventions have been shown to increase adherence, but whether they also resulted in meaningful improvement of symptoms or quality of life for the users is uncertain (Wozniak, Lasserson, & Smith, 2014).

Studies based on interviews with patients and/or their partners have explored the field and have shown the importance of context, be it within the family or society in general (Henry & Rosenthal, 2013; Luyster et al., 2016; Ward, Gott, & Hoare, 2017; Zarhin, 2015, 2018). CPAP users have been asked about their experiences with CPAP; however, Ward, Hoare, and Gott (2014) question whether the researchers’ concern about low adherence might have unduly influenced such studies by portraying CPAP treatment as inherently problematic. Therefore, the aim of present study was to further explore users’ individual experiences of CPAP use in an attempt to enrich the understanding of adherence beyond numeric measurements. The research question was as follows: How do patients with obstructive sleep apnoea experience and manage their use of CPAP?

## 3 THE STUDY

### 3.1 Design

A qualitative design based on individual narrative thematic interviews was used to explore patients’ experiences living with OSA and using CPAP. A narrative is constituted by events presented in a temporal order where life experiences are reconstructed and given meaning (Mattingly, 1994). Importantly, together with the events, others and context are also included in the stories. How characters are inscribed may mirror the roles individuals are “playing” in the story (Riessman, 2008), which again relate to the structure of our social world (Stephens & Breheny, 2013). When assaulted by illness, people try to create coherence between their prior and present life and self to make sense of the situation. One way of doing this is by telling one's story (Murray, 2000; Williams, 1984).

### 3.2 Setting and sample

The study was conducted in a Norwegian outpatient sleep clinic. Inclusion criteria were adults 18 years and older, diagnosed with OSA and having personal experience with CPAP use. In Norway, the Regional Health Authority provides patients with the necessary CPAP equipment for free. At the time of the study, the present clinic offered regular follow‐ups. Potential participants were approached by the nurses at a follow‐up clinic visit. Patients who expressed interest were introduced to the study and received written information regarding the purpose, the interview method, how their information would be handled and the professional background of the researchers. The recruiting nurses passed the name and contact information to the first author, who called the patients within one week. Three patients who initially expressed interest did not answer when contacted and one patient was excluded due to language difficulties.

The recruitment was performed in parallel with the interviews. We aimed to recruit a heterogeneous sample with respect to age, sex and duration of CPAP use, and both authors continuously evaluated information power (Malterud, Siersma, & Guassora, 2015) to obtain rich and varied data. Seven participants were included as follows: two women and five men, aged 36–76 years. Their self‐reported duration of CPAP use ranged from four months to three years. They all lived with a partner, except one participant who was in a relationship but lived alone. One had children living at home. The oldest participant was retired, while the other six worked.

### 3.3 Data collection

The first contact was established over the phone. The participants chose the time and place for the interviews; three preferred their own homes, three an office at the clinic and one at the interviewer's home. The interviews took place between March–June 2017 and were performed by the first author, a female nurse with clinical experience in the field of OSA and CPAP treatment.

The duration of the interviews varied from 33–57 min, and they were digitally recorded and transcribed verbatim by the first author. Before the audio recorder was started, the interviewer informed the participant about the purpose of the study, that participation was voluntary and about their rights to withdraw from the study with no consequence to their care or treatment. An informed consent form was then signed.

In the interviews, the participants were asked to briefly introduce themselves and thereafter they were asked to tell about their experiences with OSA and CPAP. An interview guide including themes like being diagnosed with OSA, beginning with CPAP, current use of CPAP and recommendations for beginners was used as an *aide memoire*. Through the use of general, opened‐ended questions, the participants were given the opportunity to talk freely. The interviewer had education in motivational interviewing (MI), and elements of this technique were used to elicit the participants’ narratives. Brief notes and reflections were made shortly after each interview as reminders for analysis.

### 3.4 Ethical considerations

This study was conducted in line with the ethical principles for research outlined in the Declaration of Helsinki (World Medical Association, 2013). Research Ethics Committee approval was obtained by the Norwegian Centre for Research Data (reference 52103/3/AGL).

The participants were informed that the interviewer worked as a nurse at the clinic, but none of them had met her in a therapeutic role. After the interviews, the participants could contact the primary researcher in case of questions or matters they wanted to discuss further. In this article, names, personal characteristics and places were changed to protect the participants’ anonymity.

### 3.5 Data analysis

Initially, the first author read the transcripts of each interview several times to become familiarized with the data and to obtain a general impression of the interviews’ content. The participants placed CPAP use in a larger “story” about their life including past and present. To further explore those stories, significant events were identified and placed in a temporal order influenced by narrative analysis (Riessman, 2005, 2008). Each interview was ordered in the following themes: before knowing of OSA, turning point and current CPAP use. The events were coded and thereafter clustered in more abstracted categories across the interviews, which led to the construction of two main narratives in response to the research question.

### 3.6 Rigour

Using a qualitative design demands an understanding of research as the result of an interaction of the participant(s), researcher(s) and context (Finlay, 2002). Transparency was ensured by a careful description of the study, the correspondence of the analysis to the data material and the research process. In an attempt to provide trustworthiness and coherence (Riessman, 2008), the first and second author met regularly and critically questioned their own influence on data production and interpretation. Furthermore, the steps in the analysis and preconceptions were challenged and discussed. We emphasize that what is presented in this article is not the “true” version of what the participants experienced, but our understanding and translation of what was told.

## 4 RESULTS

Overall, two main storylines on CPAP adherence were identified: we titled the first one, “A route of devotion” and the second, “A route of negotiation.” The interviews comprised rich and varied details, but at an abstract level, their narratives followed one or the other of these trajectories.

### 4.1 A route of devotion

#### 4.1.1 Life disturbed by excessive daytime sleepiness

For Alyssa (female, in her fifties), Brian (male, in his fifties) and Carl (male, in his forties), life before CPAP was described as a struggle. With excessive daytime sleepiness, participating in social activities and keeping up at work was challenging. Alyssa, who worked in a kindergarten, explained:

I brushed my teeth in the morning, looked in the mirror and thought, “This will be a hard day.” It was bad. When I lay with the children for their nap, I lay and concentrated on the pattern on the mattresses to not fall asleep.

Their poor sleep caused negative effects on their health. They talked about “feeling it in the heart” and “a huge strain on the body at night.” Additionally, the snoring and apnoeas affected the sleep of their bedpartners. This generated both worry and irritation, as for Brian's wife:

I did not sleep well, and it was very stressful. I often woke up during night and had to go to the bathroom. Went back to bed and snored like hell—excuse the expression. My wife was not happy with that as I kept her partially awake and she pulled the duvet and pushed me in the side.

#### 4.1.2 A relief knowing the cause of the problem – something fixable

Seeking medical help and being told of their OSA was generally described as a relief, since not knowing what was wrong was an extra burden. Carl had worried that he was burned‐out at work, or that “something more serious” was going on. He said:

Then she [the MD] sent me to a test and they found out that this was not so good. However, it was good news for *me* because that meant there was something that could be fixed.

Even though the diagnosis put the pieces in place, for some it was nevertheless a shock at first, as OSA threatened life itself and familiar ways of living. Alyssa told:

Getting the results, I got shocked because I did not think it was that bad. He [the MD] really scared me. I thought I could die of it! And when he started talking about my driving license too, I thought “What?! Is it that bad? It cannot be! He must have got the results wrong.”

As untreated OSA was intrusive in everyday life, anything that could improve the situation was worth trying. Brian who described his daytime functioning as “freaking terrible” hoped for a change, even if he at first did not know what to expect:

Brian: The motivation is that you get a much better life. Absolutely 100% for sure.

Interviewer: But you did not know that when you first started …

Brian: Not at all. I really had resistance to it at first. I thought “A machine and tubes and everything.” At that point, I had surrendered. Then it was just “Bring it on and let’s see what it is.”

#### 4.1.3 CPAP becomes a natural part of daily living

The first phase of using CPAP was characterized by “learning by doing.” In spite of having some initial troubles with technicalities related to the CPAP, they expressed gratefulness because they felt better using the device. Putting the mask on every night became “a ritual and a new normality, almost like brushing your teeth” as Carl said. However, this did not happen immediately; it was a process described as “practicing” and “making decisions.” The thought of giving up never occurred to them, as the alternative – a disturbed daily life – was worse. In addition to feeling poorly and unwell, other things were at stake, as for Alyssa:

I thought “This has to work out. I cannot lose my driver’s license.” So, there was no way around it, it was just getting on with it and I was the one who had to do something.

Their sleep quality and daytime functioning before CPAP treatment provided the benchmark from which they undertook the necessary adjustments needed to adapt, accept and include CPAP in their everyday lives. The positive effects on sleepiness and illness were the strongest motivational factor. They knew that untreated OSA could cause other health problems and they emphasized both the importance of regaining wellness in the present and maintaining good health for the future. As disturbances from snoring and restless sleep were reduced by the use of CPAP, their bedpartners also benefitted. CPAP use was therefore described as a win–win situation for everybody. Based on their positive experiences, they defined themselves as spoke‐persons for CPAP. They encouraged others to either get a sleep study or commit to CPAP. They were concerned for those who did not manage to use CPAP and balanced their desires to both promote its positive impacts and let each person find out for oneself.

Using CPAP became “a good thing” and an easy choice, as they transitioned from a state of feeling drained, into a state of wellness portrayed as a new existence. This was described as “life‐changing” and “dreamlike.” With their positive treatment outcome, CPAP was an obvious part of their lives and they reported using CPAP every night.

The participants following this path portrayed themselves as persons taking action and taking responsibility for their own health and well‐being. For them, CPAP was a solution to their health problems, and thus, they were devoted to using CPAP. Therefore, we refer to these participants as “the devotees.”.

### 4.2 A route of negotiation

#### 4.2.1 Ordinary life with manageable tiredness

For Diane (female, in her fifties), Eric (male, in his thirties), Fabio (male, in his sixties) and George (male, in his seventies), the symptoms and signs before being diagnosed with OSA had been either absent, mild or difficult to interpret. George, suffering from other long‐term conditions, attributed his tiredness to that. Diane remembered being somewhat tired but thought that this was quite normal. Eric had poor sleep, but none of his previous check‐ups had so far revealed anything abnormal. For Fabio, there had been no symptoms whatsoever. He recalled:

I did not notice anything myself. I was told that I snored, and my family had noticed apneas, but this was not a problem to me and you don’t do much about it when it’s not a problem.

Their reasons for seeking medical help came mostly out of being made aware by others. For example, concerned family members witnessed snoring and apnoeas, or doctors recognized diffuse symptoms as signs of a potential problem. Diane had trouble understanding and accepting that she had OSA. She felt and was told by others that she did not fit a preconceived notion of an OSA patient as a sleepy, snoring, elderly and overweight man:

It was hard to put myself in a category; I did not quite find mine [my category]. […] I thought “I have to accept this,” but I notice how I’m not about to accept it, so … it’s not entirely in place.

#### 4.2.2 Many things to consider – ambivalence towards CPAP use

With diffuse or non‐existent symptoms, OSA was rather abstract to them and for some this led to ambivalent thoughts about the diagnosis and the need for treatment. Starting to use CPAP also had a negative impact on their self‐image. Fabio, who had previously felt healthy, found himself suddenly diagnosed with OSA accompanied by a serious heart condition; for Diane, who knew of elderly family members using CPAP, the device reminded her of getting older. Additionally, she felt less feminine, both as a snorer and when wearing the CPAP mask and she pictured herself being “almost depressed” wearing the mask in the beginning. Eric related his reluctance to use CPAP to the lifelong commitment he allegedly had to make:

In the beginning, I think some of the problem was that I didn’t want to. I didn’t want to give up […]. They said: “There’s no cure for this, you’ll just have to use this machine.” To sleep with CPAP for the rest of my life … I really didn’t want to.

The device and mask were often described as big and interfering. With issues such as other health conditions to deal with, avoiding facial marks in the morning, or not wanting to wear the equipment in front of others, its “job” of treating OSA was sometimes lost. Their ambivalence towards CPAP resulted in negotiations about its use, depending on the specific situation they were in:

Diane: I haven’t used it [the CPAP] when my boyfriend sleeps over. It stands there, but I don’t use it.

Interviewer: You have it on the nightstand? You don’t put it away?

Diane: No. That’s what I thought I would do in the beginning. I thought “I’m going to live alone for the rest of my life!” […] If there was a new man in my life, I think I had hidden it [the CPAP] and then gradually said something about this.

As the immediate perceived benefits of using CPAP varied, their motivation was instead focused on preventing future illness. CPAP use became a negotiation between the present discomforts caused by the CPAP and the wish to maintain good health in the future. George, who reported having poor health in general, endured CPAP use in the hope of an occasional good night's sleep that provided him with what seemed like superpowers. He told:

I do have good nights where I sleep well and wake up refreshed, completely refreshed and I think: “Gee, now I can do almost anything!”

The concern from significant others was one reason to continue with CPAP. For example, Fabio largely used CPAP for the sake of his family, as he did not experience any positive effects himself. Talking about what kept him going with CPAP after almost three years, he said:

What can I say about that? I guess it’s … I am told to use it, both by my girlfriend and my son … but no … I do not really know. It is not easy for me to say … I have it [the CPAP], so I guess I have to use it.

Even if CPAP was part of their lives, the hope of one day not needing it was present. Making the choices about its use from day to day based on the context, rather than signing up for a lifelong commitment, made it easier. Regardless of their ambivalence to CPAP, they all would advise those new to CPAP to give it a try. Fabio summarized:

Although there may be some challenges, just go for it. Finally, it becomes part of everyday life.

These participants inscribed themselves in this narrative as people who were engaged in making and remaking decisions about their use of CPAP based on the context. They were actively resisting the device as a symbol of illness, but at the same time, weighing presents discomfort against the risk of future illness and their concern for others. The negotiations were more or less ongoing, and thus, we refer to these participants as “the negotiators.”

## 5 DISCUSSION

Through two storylines, we have described how the participants adjusted to their OSA diagnosis and adhered to CPAP treatment in different ways. Even though the details differed, their experiences can be shared via two narratives – one portraying a route of devotion and the other of negotiation.

When illness encroaches on one's life, familiar ways of acting, feeling and thinking are challenged. Illness may also cause a rupture in relationships between the self and others; new habits need to be learned and new meanings have to be found to preserve former roles and social interactions (Charmaz, 1991, 1995, 2002; Vann‐Ward, Morse, & Charmaz, 2017; Williams, 1984). During the interviews, the participants organized their experiences according to time. To make sense out of personal experiences, a narrative's structure, where one thing happens as a consequence of another, is essential (Mattingly, 1994). Stories told are not isolated anecdotes, but rather the result of merging past and present to make meaning (Riessman, 2008). To make CPAP use meaningful, the participants placed the treatment into their life history; thus, it was not possible to see their use of CPAP as unrelated to their life situation. Earlier studies have shown CPAP users’ stories of “pay‐offs” and “trade‐offs” relating to the benefits and sacrifices of CPAP use in comparison with their prior life situation (Dickerson & Aku‐Zaheya, 2007; Ward et al., 2017), and we interpret this to also be a way of placing CPAP into a lifetime perspective, similar to what the participants in this study did.

With their own life situation as the backdrop, the participants were mostly satisfied with what they had accomplished with respect to CPAP even though their use differed. Individuals wish to locate themselves in an understandable context to find meaning, and their actions need to be comprehensible as well as providing a desired outcome (Mattingly, 1994). For the devotees, it made sense to use CPAP every night as they regained their health and their daytime life quality. When the negotiators occasionally abandoned CPAP, they created meaning as they perceived CPAP use as less meaningful in specific settings. This also reflected their ambivalence towards CPAP and whether to use the mask or not. The devotees on the other hand expressed no ambivalence, as abandoning CPAP was not considered to be a viable option for them. This contrasts from the findings of Zarhin and Oksenberg (2017), where ambivalence was present among both non‐users and users regardless of CPAP's perceived effect. Additionally, Zarhin and Oksenberg (2017) pointed to subgroups of users related to levels of adherence which correlate to our findings. The devotees in the present study probably correspond to what they refer to as “adherent users,” whereas the negotiators resemble those they refer to as “partially adherent users.” In an article on adherence with diabetes care, Lutfey and Wishner (1999) also discussed alternative perspectives, other than just labelling patients as “noncompliant” when not doing as recommended. Based on the findings from the present study, where CPAP use was strongly context‐related, we share such thoughts, as we do not interpret occasionally skipping CPAP as being non‐adherent, but rather as a natural way of adapting their CPAP use into usual everyday living and preferred roles.

Adapting to the new context, where one's familiar self should merge with the new diagnosed, CPAP‐using self, was sometimes challenging for the negotiators. The mask partly symbolized unattractiveness, old age and sickness and by not using CPAP each night they could shift their perspective from badness to wellness to maintain their known self. Such a shift is not a matter of doing right or wrong, but rather a way of coping and maintaining control of one's own life situation and self when living with a long‐term condition (Paterson, 2001). For the devotees on the other hand, there was just one shift, as using CPAP returned them to their preferred self, at least during the daytime. The influence of both OSA and CPAP on the self was also shown in Zarhin's (2018) study, pointing to how both OSA and CPAP can cause a feeling of disability and an experience of becoming a social deviant. Similar findings were described by Ayow, Paquet, Dallaire, Purden, and Champagne (2009), as they referred to patients feeling embarrassed and/or ridiculed, either when being sleepy in social settings or when wearing the CPAP mask. Such thoughts are not raised out of “nothing,” but rather stem from cultural norms defining the roles of healthy/ill or normal/deviant and thereby a result of social interaction (Burr, 2015). Even if these feelings are diffuse or abstract, nurses and others working close to CPAP users need to open up to such a perspective, especially as self‐management of long‐term illness includes perspectives beyond “doing” and add dimensions of “being” and “becoming” (Kralik, Koch, Price, & Howard, 2004).

When WHO (2003) addressed the challenge of adherence to long‐term therapies, they underscored how adherence is a dynamic process reflecting behaviour and also how adherence must be differentiated from compliance. Whereas the latter represents an approach simply described as “do as you are told,” adherence is depicted as teamwork between the patient and the health professionals based on the patient's assumptions. Additionally, the WHO report (2003) questioned whether it is relevant or accurate to differentiate adherence as being either “good” or “bad.” Several articles have pointed to the complex, multifaceted sides of CPAP adherence and how nurses and other health professionals need to acknowledge this to support their patients (Broström et al., 2010; Dickerson & Kennedy, 2006; Sawyer et al., 2011; Shapiro & Shapiro, 2010; Ward et al., 2017; Weaver & Sawyer, 2010). Sawyer et al. (2011) also raised a key question when addressing: Can CPAP adherence be accurately measured? In defining adherence as a processual and contextual phenomenon, our opinion is that adherence can at least not be limited to measurements of objective, downloaded data, such as hours of use per night.

### 5.1 Limitations

During the narrative analysis, our understanding of the participants’ stories gradually evolved. Holstein and Gubrium (2012) point to the varieties in narrative analysis and how a narrative's “what” (happened) differs from a narrative's “how” (are stories told). Even though the findings from the present study to some extent show how the self and identity were related to OSA and CPAP and inscribed in cultural and social norms, we acknowledge that if a more extended analysis of the “how” had been added, we might have reached an even more in‐depth understanding of the narratives.

In addition to the narratives’ “what” and “how,” Riessman (2008) also points to the aspect of “to whom” or “for what purpose.” Although the participants mostly addressed themselves to the primary researcher as a researcher rather than as a nurse, knowing that she was a colleague of the recruiting nurses could have affected the participants’ willingness to speak freely. On the other hand, her familiarity with OSA and CPAP likely helped facilitate a common language and understanding. Even though the narratives addressed experiences previously shared by other patients in clinic, the impression was that they talked differently on the subject than they would have in a clinical encounter, which could add to new understanding of what becoming and/or being a CPAP user could be, from the patients’ perspective.

According to narrative methodology, it is not intended to illuminate all circulating narratives within an area (Riessman, 2008). Therefore, data saturation, in the meaning of being exhaustive, was not a purpose presently. For example, stories from non‐users of CPAP were not explored. The credibility of the narratives developed, depends on the data richness, as argued by Malterud et al. (2015). Each interview provided rich information and therefore, the two narratives about devoted or negotiated use of CPAP seems reasonable to be trustworthy and transferable to other patients and contexts.

## 6 CONCLUSION

This narrative analysis showed that the stories of the seven participants could be portrayed as two different trajectories of CPAP use, where experiences of illness and wellness play a significant role. In the storyline called “A route of devotion,” the users experience significant daytime problems that are relieved by the use of CPAP and as they regain wellness in their lives. The storyline “A route of negotiation” describes CPAP users who experience vague or no symptoms, and thus, fewer daytime improvements related to CPAP are experienced. Rather than symbolizing a path to wellness, for these patients, the CPAP machine symbolizes illness and unattractiveness that do not align with their sense of social self. These findings suggest that adherence to CPAP must be understood in light of how patients make sense of CPAP use by connecting it to their prior and current life experiences. Taking such a perspective, a natural consequence could be to explore how CPAP fits into each patient's life. This requires listening actively to patients’ histories and being curious and focusing on the person in context rather than just the use of CPAP.

## CONFLICT OF INTEREST

No conflict of interest has been declared by the authors.
